# Tissue Discrimination by Uncorrected Autofluorescence Spectra: A Proof-of-Principle Study for Tissue-Specific Laser Surgery

**DOI:** 10.3390/s131013717

**Published:** 2013-10-11

**Authors:** Florian Stelzle, Christian Knipfer, Werner Adler, Maximilian Rohde, Nicolai Oetter, Emeka Nkenke, Michael Schmidt, Katja Tangermann-Gerk

**Affiliations:** 1 Department of Oral and Maxillofacial Surgery, Friedrich-Alexander University of Erlangen-Nuremberg, Erlangen 91054, Germany; E-Mails: Florian.Stelzle@uk-erlangen.de (F.S.); Christian.Knipfer@uk-erlangen.de (C.K.); maximilian.ro@gmx.de (M.R.); nicooetter@gmail.com (N.O.); emeka.nkenke@uk-erlangen.de (E.N.); 2 Graduate School in Advanced Optical Technologies, Friedrich-Alexander University of Erlangen-Nuremberg (SAOT), Erlangen 91054, Germany; E-Mail: michael.schmidt@lpt.uni-erlangen.de; 3 Department of Medical Informatics, Biometry and Epidemiology, Friedrich-Alexander University of Erlangen-Nuremberg, Erlangen 91054, Germany; E-Mail: Werner.Adler@imbe.med.uni-erlangen.de; 4 Bavarian Laser Center, Erlangen 91054, Germany; 5 Institute of Photonic Technologies, Friedrich-Alexander-University of Erlangen-Nuremberg, Erlangen 91054, Germany

**Keywords:** autofluorescence, fluorescence, laser ablation, laser surgery guidance, remote optical measurement, remote surgical methods, spectra analysis

## Abstract

Laser surgery provides a number of advantages over conventional surgery. However, it implies large risks for sensitive tissue structures due to its characteristic non-tissue-specific ablation. The present study investigates the discrimination of nine different *ex vivo* tissue types by using uncorrected (raw) autofluorescence spectra for the development of a remote feedback control system for tissue-selective laser surgery. Autofluorescence spectra (excitation wavelength 377 ± 50 nm) were measured from nine different *ex vivo* tissue types, obtained from 15 domestic pig cadavers. For data analysis, a wavelength range between 450 nm and 650 nm was investigated. Principal Component Analysis (PCA) and Quadratic Discriminant Analysis (QDA) were used to discriminate the tissue types. ROC analysis showed that PCA, followed by QDA, could differentiate all investigated tissue types with AUC results between 1.00 and 0.97. Sensitivity reached values between 93% and 100% and specificity values between 94% and 100%. This *ex vivo* study shows a high differentiation potential for physiological tissue types when performing autofluorescence spectroscopy followed by PCA and QDA. The uncorrected autofluorescence spectra are suitable for reliable tissue discrimination and have a high potential to meet the challenges necessary for an optical feedback system for tissue-specific laser surgery.

## 1. Introduction

The advantages of laser surgery can be diminished by the lack of tactile feedback, which is crucial for controlling the ablation depth during laser surgery. The surgeon does not receive sufficient information about the penetration depth of the laser or the tissue type being ablated at the bottom of the cut. Hence, there is a risk of iatrogenic damage or of the destruction of anatomical structures like peripheral nerves. Especially in oral and maxillofacial surgery, this may result in major functional and aesthetic restrictions.

The approach of the present study is the investigation of a fast method for tissue discrimination, for use in a real-time process control for laser surgery. Hence, no separation of tissue optical properties have been conducted in the processing of the spectra, as this would account for a higher computation time in the feedback-process. Autofluorescence spectroscopy is known to be a robust and straightforward approach to characterize physiological and pathological tissue [1–7]. Autofluorescence of tissues is caused by several endogenous fluorophores. These include fluorophores from tissue matrix molecules and intracellular molecules like collagen, elastin, keratin and NADH. Hence, the autofluorescence signal represents a superposition of diverse fluorescence emissions. Additionally, the autofluorescence signal is affected by the tissue's light scattering and absorption properties. Light scattering is affected by tissue morphology, such as nuclear size, distribution, epithelial thickness and collagen content [8]. These properties are different and specific for each tissue type. However, there is only scarce information about the possibility of differentiating physiological tissues by means of fluorescence-based spectroscopy for remote surgical guidance. To realize real-time process control for laser surgery, it is important to use diagnostic methods that are highly time-efficient. To meet this requirement, uncorrected (raw) autofluorescence spectra were used in this study for an optical, non-contact tissue differentiation.

Hence, the aim of this study was the realization of a robust remote process control for laser surgery, using information from tissue-derived autofluorescence spectra. An autofluorescence measurement setup was established to meet the conditions of surgical intervention in an operating room under clinical conditions: Measurement and analysis procedure have to reliably work even under adverse conditions, like: (I) deviations of measurement distances with concomitant change in signal intensity, (II) ambient light and (III) the affect of superficial blood contamination on optical tissue characteristics [9].

## 2. Experimental Section

### 2.1. Tissue Samples

Autofluorescence spectra were acquired from nine different *ex vivo* tissue types: Cancellous bone, cortical bone, cartilage, fat, muscle, nerve, salivary gland, mucosa and skin. The tissue types were obtained from 15 domestic bisected pig heads (Table 1). A total of 135 tissue samples were examined within six hours *post-mortem*. Tissue samples with a dimension of 4 × 4 cm and a thickness of 5–7 mm were prepared, except for the nerve tissue samples, which were prepared with a length of 5 cm and an average diameter of 1 cm, due to their specific anatomical structure. In order to prepare the tissue samples with minimal iatrogenic damage to the tissue, a surgical scalpel was used. Superficial contamination and clotted blood particles were removed through careful manual rinsing with a physiological saline solution (0.9% NaCl). The tissue samples were permanently moistened after preparation and stored in an opaque box to avoid desiccation and subsequent altering of tissue-specific properties. The process of tissue preparation as well as the measurement of the spectra was conducted under a constant room temperature of 22 °C. Tissues were checked for pathological alterations due to local or systemic diseases before measurements were taken.

### 2.2. Experimental Setup

**Figure 1.**
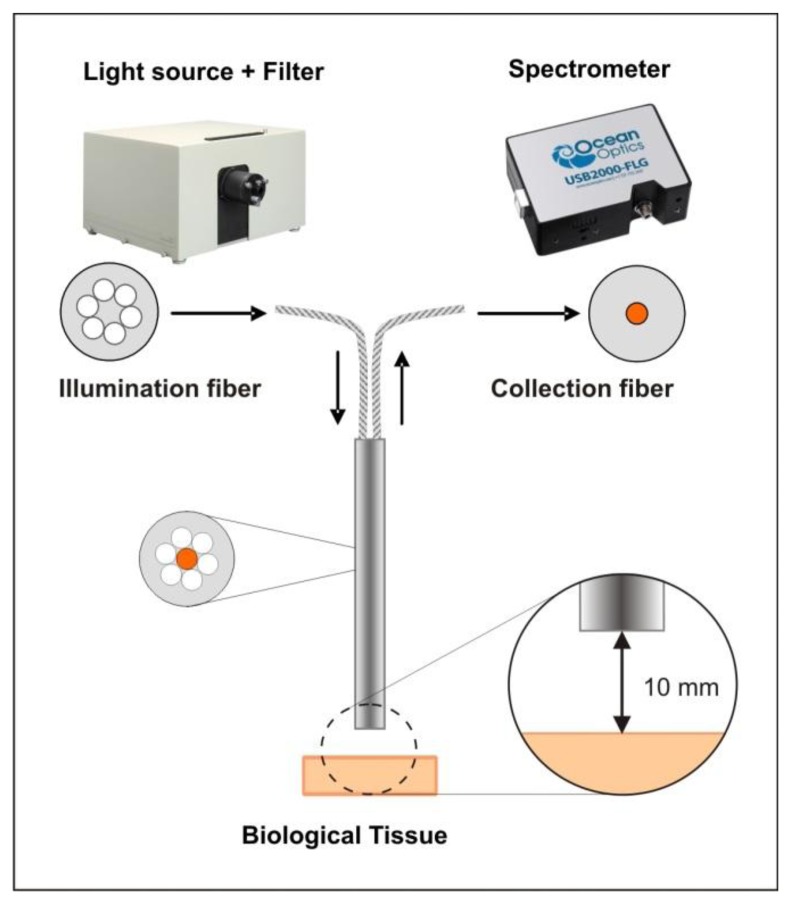
Schematics of the experimental setup.

The measurement setup (Figure 1) consisted of a light source (mercury lamp, 200 watts, Newport Spectra-Physics, Irvine, CA, USA) with a 377 nm band-pass filter for fluorescence excitation (±50 nm bandwidth) (Edmund Optics, Barrington, NJ, USA), a reflection/backscattering probe with six illumination fibers around one collection fiber with a numerical aperture of 0.22 ± 0.02 and a fiber diameter of 600 μm (QR600-7-SR-125BX, Ocean Optics Inc., Dunedin, FL, USA) and a spectrometer (USB2000-FLG, 380–1050 nm Ocean Optics Inc.) for collecting the autofluorescence spectra.

### 2.3. Autofluorescence Collection

We investigated the discrimination of nine *ex vivo* tissue types by using the uncorrected (raw) autofluorescence spectra in a wavelength range of 450–650 nm. During the measurement, each tissue sample was placed on matte black paper to avoid any reflection from the underlying surface. The excitation light spot of the probe had a diameter of 5 mm. To meet the requirements of clinical practice, the measurements were performed in contactless mode with a distance of 10 mm between the fiber probe and the tissue sample. The autofluorescence spectra were obtained from three different measurement spots on each tissue sample. The distance between the single measurement spots was 5 mm, to avoid any bias due to overlapping spots. For each measurement spot, 30 consecutive scans were recorded. Hence, a total of 1,350 spectra were obtained for each tissue type. The spectra acquisition was conducted with a computer working with the Spectra Suite spectrometer software from Ocean Optics, Inc. The following software settings were used: (1) integration time 4 ms; (2) scans to average 1 and (3) boxcar width 1. The experiments were performed under laboratory conditions in a dimmed environment with residual stray light. The ambient light was subtracted from the fluorescence spectra through a spectrometer correction function. Complete darkness was avoided, as such laboratory conditions would not meet the practical requirements for further clinical studies.

To ensure the reproducibility of the measurements, the power of the excitation light source was checked and the wavelength was calibrated using a spectral calibration lamp.

### 2.4. Data Processing

For further statistical analysis, a wavelength range from 450 nm to 650 nm was considered. The excitation wavelength covered a range from 327 nm to 427 nm. Because the emission wavelength should start at least 10 to 20 nm away from the excitation wavelength, data analysis starts from 450 nm. Over 650 nm, no prominent autofluorescence emissions were observed. The raw acquisition data were centered—for each of the wavelength measurement values, the mean of all measurements (at each specific wavelength) was subtracted from the measurement value. After pre-processing, the spectra used included 552 data points between 450 nm and 650 nm.

### 2.5. Statistical Analysis

We determined the tissue type of each single measurement *via* quadratic discriminant analysis (QDA) [10]. Prior to training the QDA classifier, we reduced the number of variables through principal component analysis (PCA) [10]. This method determines so-called principal components, which are linear combinations of the original variables that are responsible for a decreasing part of the total scatter of all data, so that the first principal component is responsible for the largest amount of variability of all data. The second principal component describes the second largest amount and so on. Thus, a variable reduction is possible, although a very high amount of variation in all data can be described, e.g., using the first ten principal components. To determine the classes of all observations in a fair way, we performed leave-one-out cross validation. Using this method, observations of all but one pig were used to train the PCA and then perform a PCA transformation of these data. In an inner cross-validation loop, the optimal number of principal components for training the QDA was determined. This optimal number was then used to train a QDA. The tissue types of the observations of the pig excluded in the first step were predicted by calculating the determined PCA transformation of the measurements and then performing a QDA prediction, using the trained method. Leave-one-out cross validation means that we iterated this process for all pigs, so that we had predictions of tissue types of all measurements in the end. The classification performance was evaluated by using receiver operating characteristic analysis (ROC) for all pairwise tissue type comparisons, *i.e.*, for example for the question how well fat was discriminated from nerve tissue. In an ROC curve, sensitivities and specificities for all possible thresholds are calculated and plotted, where the threshold determines the class membership (the tissue type) of the observation depending on the predicted class probabilities, which was calculated using the QDA. The optimal cutpoint for each pair of tissues (e.g., fat *vs.* nerve) is defined as the cutpoint maximizing the combination of sensitivity and specificity given in the Youden index (sensitivity + specificity − 1). All statistical calculations were performed using the statistical programming language R V2.13.1 [11], with the ipred V0.8-11 [12] and Daim V1.0.0 [13] packages.

## 3. Results and Discussion

### 3.1. Results

**Figure 2.**
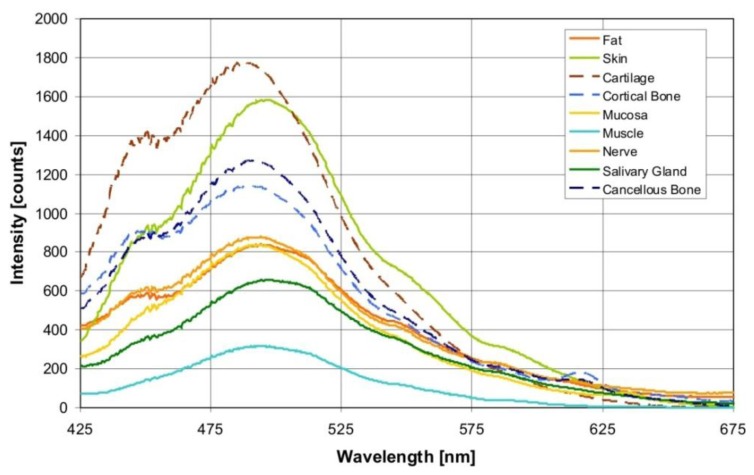
Autofluorescence spectra (averaged over 900 measurements per tissue type), excitation wavelength: 377 ± 50 nm).

Figure 2 shows the averaged autofluorescence spectra collected from the investigated tissue types (averaged over 900 measurements per tissue type). The measured autofluorescence spectra showed a superposition of broad emission peaks in the visible wavelength range with a maximum at 440–450 nm, and at 490 nm.

**Figure 3.**
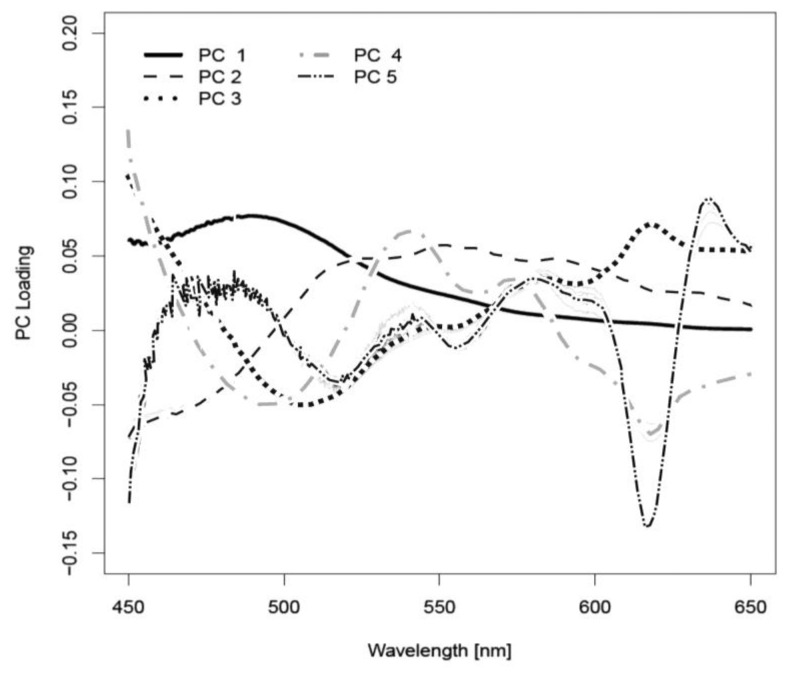
Averaged loadings of the principal components PC1–PC5 determined in 15 cross-validation runs.

As described earlier, the PCA transforms the data (autofluorescence measurements of all wavelengths) into linear combinations, so-called principal components (PCs). Depending on the cross-validation run, *i.e.*, which pig was excluded when a PCA was calculated, 15–30 principal components (PCs) were determined to be optimal for tissue differentiation using QDA. For each principal component, so-called loadings are calculated. These loadings represent the coefficients that are used when measurement values of all wavelengths are combined to form the PC. Averaged over all 15 cross-validation runs, the first five PCs were found to be responsible for 99.9% of the data variation. The first principal component (PC1) is responsible for the greatest part of the variation. It described 96.0% of the variance of the autofluorescence spectra. The loading curve of PC1 was characterized by a broad peak with a maximum at about 480–500 nm. The contribution of PC2 and PC3 to the variance was 3.2% and 0.6%, respectively. The influence of hemoglobin was recognizable in the loading curve of PC4. Two peaks were distinguishable at 540 and 580 nm. However, PC4 was only responsible for 0.1% of the variance between the autofluorescence spectra of different tissue types. PC5 contributed 0.04% to the optical variance of the tissue types. Figure 3 shows the loadings of the first five principal components averaged over all cross-validation runs. These principal components are determined using observations of all but one pig and determine the PCA transformation of the data.

In total, 94.8% of the observations were classified correctly. Nerve was classified correctly in 96.7% of cases and it was falsely recognized as fat in only 42 cases and as mucosa in three cases. Muscle was misclassified most often—the classification accuracy was 90.9%. The sensitivity for nerve discrimination in the multiclass analysis yielded values between 96% and 100%. For the discrimination of nerve *versus* cancellous bone, the sensitivity was 100%. The specificity of tissue differentiation demonstrated between nerve and cancellous bone was 100%, between nerve and fat 96%. For salivary glands, the sensitivity *versus* all other tissues was between 96% and 100%. Areas under the ROC curve (AUC) of all pairwise comparisons between tissues are given in Table 2, sensitivities are shown in Table 3, and specificities are given in Table 4.

### 3.2. Discussion

The first approaches in tissue differentiation for laser surgery showed valuable results [14–17]. Moreover, optical methods seem to meet the needs for the discrimination of tissues through a remote feedback control system, as it does not require direct contact with the tissues. Aside from the remote measurement of autofluorescence, the diffuse reflectance method yields promising results in real-time tissue discrimination [18–23]. Our workgroup positively evaluated the prospects of diffuse reflectance as a remote controlled feedback system for laser surgery in *ex vivo* [24–26] as well as in *in vivo* [27] studies. However, the crucial nerve/fat and nerve/salivary gland tissue pairs showed reduced discrimination abilities due to the biomorphological similarity of the two tissue types in *ex vivo* experiments [24,25]. An additional reason for the implementation of a remote measurement of autofluorescence parameters is the extension of the feedback method for laser surgery of pathological tissue. Here, the method of autofluorescence is well investigated in both *ex vivo* and in *in vivo* studies [5,6,28–30]. Research in this area suggests an enhancement in the identification of pathologies when combining autofluorescence and diffuse reflectance spectroscopy [7,31,32]. These studies focus on the pre-therapeutical detection of cancer tissues as a diagnostic instrument and do not yield on the transfer of this data to an intra-operative environment. In contrast, our aim was to evaluate the viability of tissue identification in a highly time efficient and precise way by using the uncorrected (raw) auto-fluorescence spectra for a real-time process control for intra-operative laser surgery as a therapeutical tool. Other works have yet demonstrated the feasibility of an intra-operative diagnostic and therapeutic instrument concerning brain tumors [33,34]. These studies however, work with the pre-operative application of 5-aminolevulinic acid-induced protoporphyrin IX fluorescence. The present study, however, focuses on an optimization of this method for an identification of tissues without the local or systemic application of optical enhancers. This will account for an easy and practicable implementation in a real-time laser ablation feedback mechanism without any potential additional harm for the patient.

It has been proposed that the most relevant tissue-specific information is contained in its fluorophore concentrations. To reduce the effect of tissue scattering and absorption on the fluorescence signal, attempts have been made to eliminate the influence of optical tissue properties from the recorded autofluorescence signal, resulting in so-called intrinsic autofluorescence [35–38]. To estimate intrinsic fluorescence from the raw autofluorescence, the signal has to be corrected by the diffuse reflectance signal. The simplest estimation is the division of autofluorescence spectra by diffuse reflectance to a variable power [7,32,38–40]. However, to realize real-time process control for laser surgery, the focus has to be on diagnostic methods that are highly time-efficient. Since an additional measurement of diffuse reflectance to correct the raw autofluorescence signal is rather time-consuming, the diagnostic potential of uncorrected (raw) autofluorescence spectra was evaluated for optical, non-contact tissue differentiation. This approach will be beneficial for fast signal processing within the context of real-time feedback control during laser surgery. Using the raw autofluorescence spectra, we acquired information on endogenous fluorophores, and additionally on the absorption and scattering properties of the investigated tissue types. This approach offers the opportunity to realize fast data processing as well as the acquisition of additional information from optical tissue properties that may support tissue discrimination.

Using autofluorescence spectroscopy, different autofluorescence emissions for tissues can be used for tissue discrimination. To find an optimal excitation wavelength for all investigated tissue types, an autofluorescence excitation emission matrix (EEM) of the tissues was measured *ex vivo* [41]. The optimum excitation-emission for tissue differentiation (including all investigated tissue types) focused on an excitation wavelength of 370 nm. The risk of a cancerogenic impact on the tissue cells by the UV excitation light has to be considered as well. The carcinogenesis of UV radiation is primarily due to its absorption by nucleic acids. Investigations show that UV radiation at 297 nm is more than 170, 340, 560, and 2000 times as effective in killing cells as 313, 325, 334, and 365 nm radiation, respectively [42]. Hence, this hazard potential was reduced by choosing a rather safe wavelength of 377 nm for autofluorescence excitation.

Through an excitation wavelength of 377 ± 50 nm, the autofluorescence from biological tissue is evoked through various endogenous fluorophores, such as collagen, elastin, keratin and NADH, which are known to contribute simultaneously to a global fluorescence signal in the form of a superposition. In the present study, the measured autofluorescence spectra show a superposition in the form of broad emission peaks in the visible wavelength range, with a maximum at 440–450 nm, and 490 nm (Figure 2). Aside from these fluorophores, other substances in the tissue components can also contribute to the fluorescence, such as retinoids or their oxidation products in lipids. Arachidonic acid, a polyunsaturated fatty acid that is present in phospholipids, is characterized by an excitation in a spectral range of 300–370 nm and a broad emission peak at 420 up to 550 nm, and at 470 nm [30].

One major optical absorber of biological tissue in the relevant visible wavelength range is oxy- (HbO_2_) and deoxyhemoglobin (Hb). Hemoglobin exhibits a typical band structure (Soret band), which shows an oxyhemoglobin absorption maxima at 414 nm, 540 and 578 nm [9]. The influence of hemoglobin on the measured autofluorescence spectra is observable in the form of shallow dips at 540 and 580 nm (Figure 2). It was also detectable in the loading curve of PC4. However, PC4 was only responsible for 0.1% of the variance between the autofluorescence spectra of different tissue types. Hence, we suppose that for tissue differentiation, the tissue structure components play a greater role and the blood circulation plays a lesser role. However, under *in vivo* conditions, it can be possible to observe a major impact by the blood circulation. Concerning this assumption, further investigations are necessary.

In the present study, a high discrimination performance was found for the investigated tissue types (classification accuracy 94.8%). Regarding the differentiation within the nerve/fat and nerve/cancellous bone tissue pairs, the results play a crucial role in the feedback mechanism concerning bone surgery in the head and neck area. Peripheral nerves are encircled by fibrous connective tissue, which inherits a large amount of fatty tissue. Additionally, several nerve branches run through bony canals, *i.e.*, the skull base or the lower jaw—e.g., preservation of the nervus alveolaris, running within the lower jaw, is an essential factor for successful surgery in this area. These nerve branches are surrounded by a cancellous bone lamella, indicating the beginning of the bony channel. For the discrimination of nerve *vs.* cancellous bone, the sensitivity was 100%, for nerve *vs.* fat 96%. The specificity of tissue differentiation between nerve and cancellous bone demonstrated 100%, between nerve and fat 96% (Tables 4 and 5). Regarding the marginally lower sensitivity and specificity for the tissue pair nerve and fat, we assume that this is due to the bio-morphological similarity of the two tissue types [24]. Nerve fiber of the infraorbital nerve, acting as a nerve tissue sample in this study, is surrounded by a myelin sheath that contains 75% lipids (25% cholesterol, 20% galactocerebroside, 5% galactosulfatide, 50% phospholipids). On the other hand, lipocytes, the major cell population of fatty tissue, predominantly consist of lipids as well [43]. The compounds are triglycerides, cholesterol and other fatty acids [43]. Prior diffuse reflectance studies of our workgroup yielded a reduced differentiation potential with a sensitivity of 93% and a specificity of 91%, respectively [24]. However, the present study shows that autofluorescence can overcome the complicacy of reduced sensitivity and specificity in the tissue pair nerve/fat by exploiting tissue specific endogenous fluorophores. Thus, an enhancement of the discrimination ability with a sensitivity and specificity of 96% can be yielded in the crucial tissue pair nerve/fat.

The differentiation between nerve and salivary gland is of major importance for maintaining functional and aesthetic integrity during facial surgery. The parotid gland surrounds the facial nerve, which divides into five segments within that gland. Thus, it is a challenge for the surgeon to prevent damage to the nervous tissue in this area. As phospholipids are contained in salivary gland tissue as well as in the superficial layers of the nervous tissue (epineurium) [44], there is a bioptic similarity between nervous tissue and the parotid gland. Halting laser ablation at this margin due to automatic differentiation will provide secure nerve protection. Diffuse reflectance data of the tissue pair nerve/salivary gland of a prior study showed declined sensitivity and specificity with 93% and 84%, respectively [25]. However, the sensitivity and specificity for salivary gland *versus* nerve in the autofluorescence measurements yielded perfect identification parameters of 100% for both (Tables 4 and 5).

To meet the requirements of laser surgical procedures in real patients, the measurement conditions were adapted to the environment of an operation room. Complete darkness, which would avoid any bias from light sources others than the excitation light of the set-up, cannot meet these requirements. Hence, we chose a set-up with surrounding stray light to simulate an environment applicable for surgical procedures. Furthermore, a remote technique was used for the application of the excitation light and the acquisition of the autofluorescence spectra, to avoid hampering the advantages of the non-contact treatment mode of laser surgery. Hence, the measurements were performed with a distance of 10 mm between the fiber probe and the tissue surface to avoid any bias through mechanical pressure on the tissue samples. Applying measurement techniques in direct contact with the tissue is known to have an impact on optical properties in both *in vivo* and *ex vivo* studies [45–48]. Deviations in the measurement distance are not critical because of the preprocessing of spectra data. The principal components determined by PCA represent a linear combination of all wavelength intensities. Due to this reduction of the dimension, we are not concentrating on a single wavelength. The shape of the fluorescence signal remained similar in its character, even if the intensity varied. Thus, fluorescence intensity deviations within a tissue type are also not critical. This fact seems to be crucial for the application in the patient in a clinical environment.

The promising results of this study have to be considered with care concerning some limitations: *ex vivo* tissue is similar but not identical to *in vivo* tissue, due to its decreased moisture and blood content, the missing circulation and its progressive oxygenation [49,50]. Thus, further research is necessary to transfer the technique to *in vivo* tissue, taking into account the influence of blood. Despite that fact, this study demonstrated the general suitability of tissue differentiation by means of uncorrected autofluorescence spectra influenced by the scattering and absorption properties of the tissue.

## 4. Conclusions

The results of this *ex vivo* study show the high differentiation potential for various tissue types, performing autofluorescence spectroscopy followed by PCA and QDA. The uncorrected autofluorescence spectra are suitable for reliable and fast tissue discrimination. The results of this study provide a base for real-time optical guidance for tissue-specific laser surgery based on the autofluorescence spectra of bio-tissue.

## Figures and Tables

**Table 1.** Number of measurements.

**Number of Tissue Types**	**Number of Pigs**	**Number of Tissue Samples per Tissue Type**	**Number of Measurement Spots per Tissue Sample**	**Number of Scans per Spot**	**Number of Measurements**
9	15	1	3	30	12,150

**Table 2.** Confusion matrix (total error 5.20%).

	**Tissue**								

**Classified as**	**Cancellous Bone**	**Cartilage**	**Cortical Bone**	**Fat**	**Mucosa**	**Muscle**	**Nerve**	**Salivary Gland**	**Skin**
Cancellous bone	**1,348**	0	0	9	0	31	0	0	18
Cartilage	0	**1,275**	0	0	0	0	0	0	26
Cortical bone	2	0	**1,324**	0	0	0	0	59	0
Fat	0	0	0	**1,254**	16	37	42	1	0
Mucosa	0	73	1	6	**1,244**	44	3	53	0
Muscle	0	0	0	0	0	**1,227**	0	1	0
Nerve	0	0	25	81	14	0	**1,305**	2	0
Salivary gland	0	0	0	0	76	11	0	**1,234**	0
Skin	0	2	0	0	0	0	0	0	**1,306**

**Classification Accuracy**	0.999	0.944	0.981	0.929	0.921	0.909	0.967	0.914	0.967

**Table 3.** Areas under the ROC curve (AUC-ROC).

	**Cancellous bone**	**Cartilage**	**Cortical bone**	**Fat**	**Mucosa**	**Muscle**	**Nerve**	**Salivary gland**
Cartilage	1.00							
Cortical bone	1.00	1.00						
Fat	1.00	1.00	1.00					
Mucosa	1.00	0.99	1.00	1.00				
Muscle	1.00	1.00	1.00	1.00	0.99			
Nerve	1.00	1.00	0.99	0.99	1.00	1.00		
Salivary gland	1.00	1.00	1.00	1.00	0.97	1.00	1.00	
Skin	1.00	1.00	1.00	1.00	1.00	1.00	1.00	1.00

**Table 4.** Sensitivity.

	**Cancellous Bone**	**Cartilage**	**Cortical Bone**	**Fat**	**Mucosa**	**Muscle**	**Nerve**	**Salivary Gland**
Cartilage	1.00							
Cortical bone	1.00	1.00						
Fat	1.00	1.00	1.00					
Mucosa	1.00	1.00	1.00	0.98				
Muscle	0.99	1.00	1.00	0.96	0.93			
Nerve	1.00	1.00	1.00	0.96	1.00	0.99		
Salivary gland	1.00	1.00	0.96	1.00	0.96	1.00	1.00	
Skin	1.00	0.98	1.00	1.00	1.00	1.00	1.00	1.00

**Table 5.** Specificity.

	**Cancellous Bone**	**Cartilage**	**Cortical Bone**	**Fat**	**Mucosa**	**Muscle**	**Nerve**	**Salivary Gland**
Cartilage	1.00							
Cortical bone	1.00	1.00						
Fat	1.00	1.00	1.00					
Mucosa	1.00	0.95	0.99	0.99				
Muscle	0.99	1.00	0.98	1.00	1.00			
Nerve	1.00	1.00	0.98	0.96	0.98	1.00		
Salivary gland	1.00	1.00	1.00	1.00	0.94	0.94	1.00	
Skin	0.99	1.00	1.00	1.00	1.00	1.00	1.00	1.00

## References

[1] Majumder S.K., Ghosh N., Kataria S., Gupta P.K. (2003). Nonlinear pattern recognition for laser-induced fluorescence diagnosis of cancer. Laser. Surg. Med..

[2] Wang C.Y., Tsai T., Chen H.M., Chen C.T., Chiang C.P. (2003). Pls-ann based classification model for oral submucous fibrosis and oral carcinogenesis. Laser. Surg. Med..

[3] Jayanthi J.L., Subhash N., Stephen M., Philip E.K., Beena V.T. (2011). Comparative evaluation of the diagnostic performance of autofluorescence and diffuse reflectance in oral cancer detection: A clinical study. J. Biophotonics.

[4] Moro A., Di Nardo F., Boniello R., Marianetti T.M., Cervelli D., Gasparini G., Pelo S. (2010). Autofluorescence and early detection of mucosal lesions in patients at risk for oral cancer. J. Craniofac. Surg..

[5] Kamath S.D., Mahato K.K. (2007). Optical pathology using oral tissue fluorescence spectra: Classification by principal component analysis and k-means nearest neighbor analysis. J. Biomed. Opt..

[6] De Veld D.C., Witjes M.J., Sterenborg H.J., Roodenburg J.L. (2005). The status of *in vivo* autofluorescence spectroscopy and imaging for oral oncology. Oral Oncol..

[7] De Veld D.C., Skurichina M., Witjes M.J., Duin R.P., Sterenborg H.J., Roodenburg J.L. (2005). Autofluorescence and diffuse reflectance spectroscopy for oral oncology. Laser. Surg. Med..

[8] Jaques S., Alfano A.A., Fujimoto J.G. (1996). Origins of Tissue Optical Properties in the UVA, Visible, and Nir Regions. OSA TOPS on Advances in Optical Imaging and Photon Migration.

[9] Faber D.J., Aalders M.C., Mik E.G., Hooper B.A., van Gemert M.J., van Leeuwen T.G. (2004). Oxygen saturation-dependent absorption and scattering of blood. Phys. Rev. Lett..

[10] Hastie T., Tibshirani R., Friedman J. (2009). The Elements of Statistical Learning: Data Mining, Inference and Prediction.

[11] Team R.D.C. (2008). R: A Language and Environment for Statistical Computing. R Foundation for Statistical Computing.

[12] Peters A., Hothorn T. Ipred: Improved Predictors. http://cran.R-project.Org/package=ipred.

[13] Potapov S., Adler W., Lausen B. Daim: Diagnostic Accuracy of Classification Models. http://cran.r-project.org/web/packages/Daim/index.html.

[14] Kim B.M., Feit M.D., Rubenchik A.M., Mammini B.M., da Silva L.B. (1998). Optical feedback signal for ultrashort laser pulse ablation of tissue. Appl. Surface Sci..

[15] Rupprecht S., Tangermann K., Kessler P., Neukam F.W., Wiltfang J. (2003). Er: Yag laser osteotomy directed by sensor controlled systems. J. Cranio Maxillofac. Surg..

[16] Rupprecht S., Tangermann-Gerk K., Wiltfang J., Neukam F.W., Schlegel A. (2004). Sensor-based laser ablation for tissue specific cutting: An experimental study. Laser. Med. Sci..

[17] Tangermann K., Roth S., Muller D., Tragler H., Uller J., Rupprecht S. (2003). Sensor-controlled laser processes for medical applications. Proc. SPIE 5287, Laser Florence.

[18] Kienle A., Lilge L., Patterson M.S., Hibst R., Steiner R., Wilson B.C. (1996). Spatially resolved absolute diffuse reflectance measurements for noninvasive determination of the optical scattering and absorption coefficients of biological tissue. Appl. Opt..

[19] Georgakoudi I., Rice W., Hronik-Tupaj M., Kaplan D. (2009). Optical spectroscopy and imaging for the noninvasive evaluation of engineered tissues. Tissue Eng. Part B: Rev..

[20] Hidovic-Rowe D., Claridge E. (2005). Modelling and validation of spectral reflectance for the colon. Phys. Med. Biol..

[21] Bashkatov A.N., Genina E.A., Kochubey V.I., Tuchin V.V. (2005). Optical properties of human skin, subcutaneous and mucous tissues in the wavelength range from 400 to 2000 nm. J. Phys. Lond. D Appl. Phys..

[22] Ritz J.P., Roggan A., Isbert C., Muller G., Buhr H.J., Germer C.T. (2001). Optical properties of native and coagulated porcine liver tissue between 400 and 2400 nm. Lasers Surg. Med..

[23] Troy T.L., Thennadil S.N. (2001). Optical properties of human skin in the near infrared wavelength range of 1000 to 2200 nm. J. Biomed. Opt..

[24] Stelzle F., Tangermann-Gerk K., Adler W., Zam A., Schmidt M., Douplik A., Nkenke E. (2010). Diffuse reflectance spectroscopy for optical soft tissue differentiation as remote feedback control for tissue-specific laser surgery. Lasers Surg. Med..

[25] Stelzle F., Zam A., Adler W., Tangermann-Gerk K., Douplik A., Nkenke E., Schmidt M. (2011). Optical nerve detection by diffuse reflectance spectroscopy for feedback controlled oral and maxillofacial laser surgery. J. Transl. Med..

[26] Stelzle F., Terwey I., Knipfer C., Adler W., Tangermann-Gerk K., Nkenke E., Schmidt M. (2012). The impact of laser ablation on optical soft tissue differentiation for tissue specific laser surgery-an experimental *ex vivo* study. J. Transl. Med..

[27] Stelzle F., Adler W., Zam A., Tangermann-Gerk K., Knipfer C., Douplik A., Schmidt M., Nkenke E. (2012). *In vivo* optical tissue differentiation by diffuse reflectance spectroscopy: Preliminary results for tissue-specific laser surgery. Surg. Innov..

[28] Betz C.S., Mehlmann M., Rick K., Stepp H., Grevers G., Baumgartner R., Leunig A. (1999). Autofluorescence imaging and spectroscopy of normal and malignant mucosa in patients with head and neck cancer. Laser. Surg. Med..

[29] Betz C.S., Stepp H., Janda P., Arbogast S., Grevers G., Baumgartner R., Leunig A. (2002). A comparative study of normal inspection, autofluorescence and 5-ala-induced ppix fluorescence for oral cancer diagnosis. Int. J. Cancer.

[30] Bottiroli G., Croce A.C. (2004). Autofluorescence spectroscopy of cells and tissues as a tool for biomedical diagnosis. Photochem. Photobiol. Sci..

[31] Shao X., Zheng W., Huang Z. (2010). Polarized near-infrared autofluorescence imaging combined with near-infrared diffuse reflectance imaging for improving colonic cancer detection. Opt. Express.

[32] Amouroux M., Diaz-Ayil G., Blondel W.C., Bourg-Heckly G., Leroux A., Guillemin F. (2009). Classification of ultraviolet irradiated mouse skin histological stages by bimodal spectroscopy: Multiple excitation autofluorescence and diffuse reflectance. J. Biomed. Opt..

[33] Liao H., Noguchi M., Maruyama T., Muragaki Y., Kobayashi E., Iseki H., Sakuma I. (2012). An integrated diagnosis and therapeutic system using intra-operative 5-aminolevulinic-acid-induced fluorescence guided robotic laser ablation for precision neurosurgery. Med. Image Anal..

[34] Ando T., Kobayashi E., Liao H., Maruyama T., Muragaki Y., Iseki H., Kubo O., Sakuma I. (2011). Precise comparison of protoporphyrin IX fluorescence spectra with pathological results for brain tumor tissue identification. Brain Tumor Pathol..

[35] Gupta P.K., Majumder S.K., Uppal A. (1997). Breast cancer diagnosis using N2 laser excited autofluorescence spectroscopy. Laser. Surg. Med..

[36] Chang S.K., Marin N., Follen M., Richards-Kortum R. (2006). Model-based analysis of clinical fluorescence spectroscopy for *in vivo* detection of cervical intraepithelial dysplasia. J. Biomed. Opt..

[37] Schwarz R.A., Gao W., Daye D., Williams M.D., Richards-Kortum R., Gillenwater A.M. (2008). Autofluorescence and diffuse reflectance spectroscopy of oral epithelial tissue using a depth-sensitive fiber-optic probe. Appl. Opt..

[38] Volynskaya Z., Haka A.S., Bechtel K.L., Fitzmaurice M., Shenk R., Wang N., Nazemi J., Dasari R.R., Feld M.S. (2008). Diagnosing breast cancer using diffuse reflectance spectroscopy and intrinsic fluorescence spectroscopy. J. Biomed. Opt..

[39] Angheloiu G.O., Arendt J.T., Muller M.G., Haka A.S., Georgakoudi I., Motz J.T., Scepanovic O.R., Kuban B.D., Myles J., Miller F. (2006). Intrinsic fluorescence and diffuse reflectance spectroscopy identify superficial foam cells in coronary plaques prone to erosion. Arterioscler. Thromb. Vasc. Biol..

[40] Muller M.G., Georgakoudi I., Zhang Q., Wu J., Feld M.S. (2001). Intrinsic fluorescence spectroscopy in turbid media: Disentangling effects of scattering and absorption. Appl. Opt..

[41] Zam A. (2010). Optical Tissue Differentiation for Sensor-Controlled Tissue-Specific Laser Surgery. Master Thesis.

[42] Andley U.P., Lewis R.M., Reddan J.R., Kochevar I.E. (1994). Action spectrum for cytotoxicity in the uva- and uvb-wavelength region in cultured lens epithelial cells. Investig. Ophthalmol. Vis. Sci..

[43] Ross M.H., Pawlina W. (2006). Histology: A Text and Atlas: With Correlated Cell and Molecular Biology.

[44] Bowman A.S., Dillwith J.W., Madden R.D., Sauer J.R. (1995). Uptake, incorporation and redistribution of arachidonic acid in isolated salivary glands of the lone star tick. Insect Biochem. Mol. Biol..

[45] Chan E.K., Sorg B., Protsenko D., O'Neil M., Motamedi M., Welch A.J. (1996). Effects of compression on soft tissue optical properties. IEEE J. Sel. Top. Quantum Electron..

[46] Nath A., Rivoire K., Chang S., Cox D., Atkinson E.N., Follen M., Richards-Kortum R. (2004). Effect of probe pressure on cervical fluorescence spectroscopy measurements. J. Biomed. Opt..

[47] Reif R., Amorosino M.S., Calabro K.W., A'Amar O., Singh S.K., Bigio I.J. (2008). Analysis of changes in reflectance measurements on biological tissues subjected to different probe pressures. J. Biomed. Opt..

[48] Ti Y., Lin W.C. (2008). Effects of probe contact pressure on *in vivo* optical spectroscopy. Opt. Express.

[49] Salomatina E., Yaroslavsky A.N. (2008). Evaluation of the *in vivo* and *ex vivo* optical properties in a mouse ear model. Phys. Med. Biol..

[50] Palmer G.M., Marshek C.L., Vrotsos K.M., Ramanujam N. (2002). Optimal methods for fluorescence and diffuse reflectance measurements of tissue biopsy samples. Lasers Surg. Med..

